# Factors associated to neurocognitive impairment in older adults living with HIV

**DOI:** 10.1186/s40001-022-00639-6

**Published:** 2022-02-02

**Authors:** Júlia Gutierrez-San-Juan, Itziar Arrieta-Aldea, Isabel Arnau-Barrés, Greta García-Escobar, Elisabet Lerma-Chipirraz, Paula Pérez-García, Agustin Marcos, Fabiola Blasco-Hernando, Alicia Gonzalez-Mena, Esperanza Cañas, Hernando Knobel, Robert Güerri-Fernández

**Affiliations:** 1 Department de Medicina, Universitat Autònoma de Barcelona and Universitat, Barcelona, Spain; 2 Department of Geriatrics, Hospital del Mar, Barcelona, Spain; 3 Department of Infectious Diseases, Hospital del Mar Institute of Medical Research, Barcelona, Spain; 4 Department of Neurology, Hospital del Mar, Barcelona, Spain; 5 Facultat de Ciencias de la Salud y de la Vida, Universitat Pompeu Fabra , Barcelona, Spain; 6 Hospital del Mar Institute of Medical Research, Passeig Doctor Aiguader 88, 08003 Barcelona, Spain

**Keywords:** HIV, Aging, Neurocognitive disorder, Frailty, Antiretroviral therapy

## Abstract

**Objective:**

The HIV infection is a chronic disease that causes neurocognitive impairment (NI) and has been related with early development of frailty. We aimed to study the main risk factors for neurocognitive disorders and frailty in HIV older adults.

**Materials and methods:**

Cross-sectional study with 40 HIV individuals older than 65 years under antiretroviral therapy in Hospital del Mar (Barcelona) recruited between November 2019 and October 2020. Data has been obtained through clinical scores and a blood sample to evaluate NI and frailty and has been analyzed with non-parametric tests and a multivariate logistic regression model.

**Results:**

Among the 40 patients admitted for the study, 14 (35%) had positive screening for NI. We found that HIV individuals with nadir CD4+ T-cell count lower than 350 cells/mm^3^ had 39.7 more risk for NI (95% CI 2.49–632.10; *p* = 0.009). Those with a lower education level had 22.78 more risk for neurocognitive disorders (95% CI 2.13–242.71; *p* = 0.01) and suffering any comorbidity with a punctuation ≥ 1 in the Charlson Comorbidity index had an increased risk of 18.26 of developing NI and frailty (95% CI 1.30–256.33; *p* = 0.031), among them diabetes was significantly more frequent in NI.

**Conclusion:**

We observed that the main risk factors for a positive NI screening in HIV older adults were low education level, a nadir CD4+ T-cell count < 350 cells/mm^3^ and the presence of any comorbidity, highlighting diabetes among them.

## Keypoints

Aim: Which HIV older adults should be mandatory screened for neurocognitive impairment

Findings: We found in a small very well characterized cohort that individuals with lower nadir CD4+ T-cell count, meaning advanced HIV infection at any time and lower schooling are associated with higher probability of positive neurocognitive impairment test.

Message: Low nadir CD4+ T-cell count and low schooling are associated with higher risk of positive screening for neurocognitive disorder in HIV older adults

## Introduction

The human immunodeficiency virus (HIV) infection is a chronic disease suffered by 38 million people in the world in 2019 [1]. HIV is a neurotropic virus which causes central nervous system (CNS) damage and neurocognitive impairment (NI). This is found in 10–20% in early HIV stages and in 30–40% in advanced disease [2, 3]. Although the clinical range of NI associated with HIV is wide, the more severe forms have dramatically decreased due to the use of antiretroviral treatment (ART) [4, 5]. However, the milder forms continue to concern in people living with HIV (PLWH) [5–7], especially now that life expectancy is significantly longer [8, 9]. NI plays a preponderant role in the deterioration of the quality of life and functionality, carrying an accelerated appearance of frailty [10–12]. Many mechanisms for NI such as CNS virus persistence despite suppressed peripheral viral load, continuous activation of monocytes in the CNS [13–15], HIV-specific persistent inflammatory response or potential ART CNS toxicities [5, 16] have been proposed. All of these seem to be keystone in the pathophysiology of neurocognitive deterioration.

HIV-associated dementia (HAD) and HIV-associated neurocognitive disorders (HAND) have declined in the era of ART. However, the ageing of the HIV population is impacting significantly in the neurocognitive status of PLWH overlapping both conditions and posing a potential risk for frailty [10, 17, 18]. It is believed that nowadays, the NI found in HIV is linked to HIV itself but also aging-associated comorbidities such as cardiovascular disease, metabolic disorders, osteoporosis, obesity, cancer, Alzheimer disease or other types of dementias [5, 14, 19].

Neurocognitive disorders are screened using scores as the Brief Neurocognitive (BNC) or the HIV Neurobehavioral Research Center (HNRC) [20]. Among them, MOCA score has traditionally been used to the screening of NI, showing a high sensitivity (90%) but low specificity (45%). At the same time extensive tests are used to confirm those positive cases evaluating different capacities such as motor skills with Dominant hand time (GPT) or Non-dominant hand time (GPT), executive functioning with Total time (TMT-B) or verbal fluency with Phonemic (COWAT) [21]. The importance of an early diagnosis and a multidisciplinary care has been previously demonstrated [10]. However, the factors associated with NI and ageing nowadays in the HIV pandemic remain unveiled.

The aim of our study was to describe a cohort of PLWH older than 65 years under ART to determine the prevalence of a positive screening for NI associated with HIV and to analyze the risk factors for a positive result. As secondary objectives to study other components of cognition and functionality.

## Materials and methods

Cross-sectional study conducted in Hospital del Mar in Barcelona, Spain. From November 2019 to October 2020 HIV participants were selected from a clinical HIV cohort with 2094 patients followed-up by the Infectious Disease Department. Eligible participants included people over 65 years under ART. Of the 147 who reached these conditions, 40 participants were randomly selected for the study and were visited at the same day by different specialists (infectious diseases, geriatrics and neuropsychology). We excluded any patients who had a severe intellectual disability from other causes than HIV.

Demographic and clinical data were extracted from electronic medical records and clinical scores. Laboratory workups included a fasting blood draw with metabolic markers such as total cholesterol, LDL–HDL cholesterol, triglycerides, and HIV markers including CD4+ , CD8+ T-cell count and viral load.

MOCA score lower than 25/30 was considered a positive screening for NI as analysis consideration.

Schooling was categorized into three groups, lower education level including elementary school and illiterate individuals, and high school education and professional degree and university.

An integral geriatric evaluation was performed to analyze sensory impairment, sleep disorders, polypharmacy (considered as taking ≥ 5 drugs), urine and fecal incontinence, constipation, falls (≥ 2 in the last year), depressive syndrome, dementia, delirium, malnutrition and pressure ulcers. Daily life activities were assessed using Barthel and Lawton–Brody index, and nutritional status was analyzed by Mini Nutritional Assessment (MNA) screening test. Frailty was valuated with the Clinical Frailty scale and comorbidity was assessed using the Charlson Comorbidity index, which was also categorized in no comorbidities, medium–low (1–2 comorbidities) or high (≥ 3 comorbidities). All included individuals had a VACS index at baseline [15].

## Statistics

Continuous variables were expressed as medians and interquartile range (IQRs) and were compared using Mann–Whitney *U* test. Categorical variables were expressed as frequencies (percentages) and were compared with the *χ*^2^ test. A multivariable logistic regression model was fitted to analyze the positive screening for NI adjusting by age, nadir CD4+ T-cell count, educational level and comorbidity, to determine odds ratios (ORs) and 95% confidence intervals (CI) for covariates. Those variables with *p *value < 0.05 in univariate analysis were included into the multivariate logistic regression model. STATA/MP V.14.2 software.

## Results

Among 40 patients recruited for the study, 14 (35%) had positive screening for NI. Table 1 shows the cohort’s baseline characteristics (overall), and the comparison between positive and negative screening in the MOCA test. Individuals testing positive in the NI screening had lower educational level (21.43% vs 73.08%; *p* = 0.002), while individuals with university studies or a professional degree were less likely to test positive in the NI screening (42.86% vs 7.69%; *p* = 0.008) and (14.29% vs 0%; *p* = 0.048), respectively. Likewise, individuals with diabetes mellitus tested positive to NI screening (26.92% vs 0%; *p* = 0.033). Interestingly, we found no differences between groups with respect to age, gender, toxic habits or housing status (Table 1).

**Table 1:** Baseline characteristics of the cohort

	Overall	Negative screening	Positive screening	*P* value
*n* (%)	40	26 (65)	14 (35)	–
Age				0.472
Median age (IQR)	70 (68–75)	71 [67.5–77.3]	70 [68.5–75]	
< 70 years (*n*, %)	17 (42.5)	6 (42.86)	11 (42.31)	
70–80 years (*n*, %)	19 (47.5)	6 (42.86)	13 (50)	
> 80 years (*n*, %)	4 (10)	2 (14.29)	2 (7.69)	
Gender				0.178
Men (*n*, %)	34 (85)	14 (100)	20 (76.92)	
Women (*n*, %)	4 (10)	0	4 (15.38)	
Transsexual (*n*, %)	2 (5)	0	2 (7.68)	
Schooling				0.002
Elementary school (*n*, %)	24 (60)	3 (21.43)	21 (80)	
High school/professional (*n*, %)	8 (20)	5 (35.72)	3 (12)	
University (*n*, %)	8 (20)	6 (42.86)	2 (8)	
Home				0.471
Alone (*n*, %)	16 (40)	6 (42.86)	10 (38.46)	
Partnered (*n*, %)	17 (42.5)	7 (50)	10 (38.46)	
Sharing room (*n*, %)	7 (17.5)	1 (7.14)	6 (23.08)	
Toxic habits				0.534
Smoking (*n*, %)	9 (22.5)	3 (21.43)	6 (23.08)	
Ex-smoking (*n*, %)	16 (40)	6 (42.86)	10 (34.46)	
Alcohol (*n*, %)	18 (45)	6 (42.86)	12 (46.15)	
Recreation drugs (*n*, %)	6 (15)	3 (21.43)	3 (11.54)	
Comorbidities				
Hyper blood pressure (*n*, %)	20 (50)	8 (40)	12 (60)	0.507
Dyslipidemia (*n*, %)	24 (60)	10 (41.67)	14 (15.6)	0.279
Diabetes mellitus (*n*, %)	7 (17.5)	0	7 (26.92)	0.033
Chronic kidney disease (*n*, %)	3 (7.5)	2 (66.67)	1 (33.33)	0.232
Stroke (*n*, %)	3 (7.5)	2 (14.29)	1 (3.85)	0.232
Overweight (BMI 25–30) (*n*, %)	19 (47.5)	7/50)	12 (46.15)	0.816
Obesity (BMI ≥ 30) (*n*, %)	10 (25)	3 (27.27)	8 (72.73)	0.528
Liver cirrhosis (*n*, %)	1 (2.5)	1 (7.14)	0	0.168
Vitamin D deficiency (*n*, %)	15 (37.5)	5 (35.71)	10 (38.46)	0.864
Osteoporosis (*n*, %)	2 (5)	0	2 (7.69)	0.287
Pulmonary disease (*n*, %)	5 (12.5)	0	5 (19.23)	0.079
Cancer active (*n*, %)	2 (22.22)	1 (25)	1 (20)	0.858
Cancer cured (*n*, %)	7 (77.78)	3 (75)	4 (80)	0.858
Neurocognitive impairment (*n*, %)	1 (2.70)	0	1 (4.17)	0.456
Co-infections				0.634
Toxoplasma (*n*, %)	1 (2.5)	0	1 (3.85)	
Atypical mycobacteria (*n*, %)	1 (2.5)	0	1 (3.85)	
Varicella-zoster virus (*n*, %)	2 (5)	2 (14.29)	0	
Herpesvirus (*n*, %)	2 (5)	1 (7.14)	1 (3.85)	
Hepatitis C virus (SVR) (*n*, %)	4 (100)	1 (100)	3 (100)	
Hepatitis B virus (*n*, %)				
Active	1 (12.5)	0	1 (14.29)	
Controlled	4 (50)	1 (100)	3 (42.86)	
Past	3 (37.5)	0	3 (42.86)	
Invasive CMV (*n*, %)	1 (2.5)	0	1 (3.85)	
Kaposi’s sarcoma (*n*, %)	2 (5)	1	1	
Tuberculosis (*n*, %)	5 (12.5)	1 (7.14)	4 (15.38)	
HIV data				
CD4+ T-cell count nadir (IQR)	352 [163–520]	526.5 [352–603]	334 [150–400]	0.01
CD4/CD8 at diagnosis (IQR)	0.34 [0.27–0.59]	0.5 [0.29–0.65]	0.32 [0.19–0.48]	0.18
CD8+ count at diagnosis (IQR)	770 [661–1197.5]	1027.5 [800–1198]	731 [526–1197]	0.160
Zenit viral load (IQR)	33,765 [8900–90434]	55,048.5 [6050–174354]	33,000 [11000–84248]	0.632
Time since diagnosis (years) (IQR)	22 [14–27.5]	23.5 [15–29]	21.5 [13–27]	0.560
Time under ART (years) (IQR)	19 [9–22.5]	19.5 [8–24]	19 [10–22]	0.638
Time undetectable (months) (IQR)	163 [60–228]	79 [71–207]	171.5 [60–228]	0.601
Geriatric evaluation				
Barthel index (IQR)	100 [100–100]	100 [100–100]	100 [100–100]	0.768
Lawton and Brody index (IQR)	8 [8–8]	8 [7, 8]	8 [8–8]	0.479
Charlson comorbidity index (*n*, %)				0.031
No comorbidity (0)	25 (62.5)	12 (85.71)	13 (50)	
Medium–low (1–2)	10 (25)	1 (7.14)	9 (34.62)	
High (> 3)	5 (12.5)	1 (7.14)	4 (15.38)	
MNA screening (IQR)	14 [12–14]	14 [12.5–14]	13 [12–14]	0.188
Sensory impairment (*n*, %)	17 (45.95)	6 (46.15)	11 (45.83]	0.985
Sleep disorder (*n*, %)	4 (10.81)	2 (15.38)	2 (8.33)	0.510
Polypharmacy (*n*, %)	24 (64.86)	10 (76.92)	14 (58.33)	0.258
Urine incontinence (*n*, %)	4 (10.81)	1 (7.69)	3 (12.5)	0.653
Fecal incontinence (*n*, %)	2 (5.41)	0	2 (8.33)	0.285
Constipation (*n*, %)	3 (8.11)	1 (7.69)	2 (8.33)	0.946
Falls (*n*, %)	5 (13.51)	4 (30.77)	1 (4.17)	0.024
Depressive syndrome (*n*, %)	4 (10.81)	2 (15.38)	2 (8.33)	0.510
Clinical frailty scale (IQR)	2 [2–2]	2 [1, 2]	2 [2–2.5]	0.241
SPPB total (IQR)	10 [9–11]	9 [8–10]	11 [9–11]	0.400
VACS index points (IQR)	35 [33–43]	33 [33–37]	43 [33–49]	0.029
Neurological evaluation				
MOCA test (IQR)	22.37 [20–26.5]	27 [26–28]	20 [18–22]	0.000
Blood test				
Total cholesterol (IQR)	165.5 [144–188.5]	177 [150–189]	164.5 [142–186]	0.954
LDL cholesterol (IQR)	105.5 [90.5–117]	100 [78–110]	106 [95–118]	0.410
HDL cholesterol (IQR)	44.2 [34.55–55.2]	45.7 [37.7–55]	43.4 [32.8–55.4]	0.798
Triglycerides (IQR)	133.5 [96.5–183]	128 [82–188]	138 [98–169]	0.887
CD4+ T-cell count (IQR)	636.5 [477–783.5]	758 [547–958]	584.5 [459–721]	0.088
CD8+ T-cell count (IQR)	798 [579–1058.5]	968 [795–1128]	721 [561–999]	0.083
CD4/CD8 (IQR)	0.76 [0.59–1.05]	0.76 [0.58–1.03]	0.77 [0.6–1.09]	0.186
Undetectable (%)	100	100	100	–

Differences between older adults with positive and negative screening for neurocognitive impairment

*BMI* body mass index; *SVR* sustained viral response

When focusing on HIV characteristics there were no differences in time to exposure to HIV, nor in time undetectable (with no viral load detectable in blood) (Table 1). Those individuals testing positive for NI had significantly lower nadir CD4+ T-cell count [(526.5 cells/mm^3^ (352–603) vs 334 (150–400); *p* = 0.001)] and a trend to lower actual CD4+ T-cell count [(758 cells/mm^3^ (547–958) vs 584.5 (459–721); *p* = 0.088)]. Moreover, VACS index revealed statistically significant results according to NI, detecting higher scores in those patients with positive screening (33 vs 43; *p* = 0.029).

One patient was detected with neurocognitive impairment prior to the start of the study. Regarding to geriatric syndrome results are shown in Table 1. Twenty-five individuals (62.5%) had no comorbidity according to the Charlson Comorbidity index and the median punctuation in the Clinical Frailty scale was 2 (2–2). Only five patients referred having suffered ≥ 2 falls in the last year. Those who tested positive for NI were more likely to have any comorbidity (12 (85.71%) vs 13 (50%); *p* = 0.026).

In a multivariate logistic regression model to predict a positive screening for NI (Table 2), the OR for nadir CD4+ T-cell count less than 350 cells/mm^3^ was 39.7 (95% CI 2.49–632.10; *p* = 0.009), the OR for lower education level was 22.78 (95% CI 2.13–242.71; *p* = 0.01) and the OR for any comorbidity (≥ 1 in Charlson Comorbidity index) was 18.26 (95% CI 1.30–256.33; *p* = 0.031).

**Table 2:** Logistic multivariable regression

	Odds-ratio	95% CI
Age	0.91	0.75–1.11
Nadir CD4+ T-cell count (< 350 cells/mm^3^)	39.70	2.49–632.10
Education level (lowest level)	55.28	1.34–452.71
Charlson index (≥ 1 comorbidity)	18.26	1.30–256.33

Predictors of neurocognitive disorders

## Discussion

We report a significant association between having a positive screening for NI and nadir CD4+ T-cell count, lower education level and having any comorbidity among HIV+ older adults. These results, although expected, help to understand the potential underlying causes of NI among HIV individuals. Moreover, we found that the frailty phenotype was not directly associated with NI.

In our series, a third of the individuals had a positive screening for NI. This proportion is similar to prior reported studies, where 30–50% of older adults living with HIV had HAND [21]. Usually, the onset of this condition consists on subacute neurological symptoms such as lack of focus or impaired motor fine skills [6, 22]. This NI might be associated with poorer quality of life and shorter life expectancy [5, 9, 23, 24]. An early diagnosis is keystone to identify subjects at risk and to plan appropriate management strategies. In this scenario screening tests such as MOCA might help to identify individuals that could need further studies [25, 26].

We found that HIV status at diagnosis, schooling degree and comorbidities are determinants in the odds of testing positive in the NI screening. However, we also expected to report an association between aging and NI, but we did not find it. One potential explanation might be the small sample size of this cohort. But also that there might be a selection bias. Individuals who were able to participate into the study were in a good health condition, at least enough to come to the clinic and participate in the study. However, prior studies have widely demonstrated the association between age and NI [9, 11, 12, 16, 24, 27].

The association found between NI and low CD4+ T-cell nadir seems to be associated with some characteristics of the HIV infection and how it affects the immune system. Chronic HIV infection and aging are related in a state of immunosenescence which contributes to earlier appearance of comorbidities among HIV individuals [2, 3, 9, 11, 12, 17]. However, there is a subpopulation of individuals among people living with HIV known as late presenters with particularly low nadir CD4+ T-cell count. We found that individuals with a CD4+ T-cell nadir lower than 350 cells/mm^3^ had 39.7 more risk for NI. Low CD4+ T-cell nadir has been shown to be a relevant to a worse prognostic for other HIV but also for other non-AIDS comorbidities [2, 7, 9]. Nadir CD4+ T-cell count is associated with a greater destruction of the immune system by the HIV itself. Therefore, a low CD4+ T-cell count may lead to a higher viral replication, and subsequently to an increased inflammatory response, who has been claimed as responsible for many comorbidities associated to HIV. The HIV infection impairs the homeostasis of CD4 lymphocytes leading to a CD4+ destruction by direct cytopathic effect, and hyperactivation and depletion of the immune system that has been associated with multiple effects such as NI [2, 6, 9, 14]. However, likely the viral replication in reservoirs, as the brain, might have a role in the progressive deterioration observed in individuals prior to the appearance of the highly effective antiretroviral therapy [2]. The late diagnosis of the HIV infection implies low CD4+ T-cell count and, at the same time, more time of exposure to a highly replicant virus. The long term impact of this period regardless of the later time undetectable might be a potential explanation to the association observed between nadir CD4+ and neurocognitive impairment.

In the same line, schooling has also an impact in NI. Moreover, HIV patients who have low education attainment carry higher risk of developing HAND compared with HIV patients with university studies or professional degrees [28–30]. This could be related to a greater development of neuronal capacity during childhood and greater neurological training throughout life, which could act as a protective factor against the development of neurocognitive disorders [13, 28, 31]. Besides, the exposure to HIV might have impaired the normal development of the neuronal capacity. These individuals have been exposed to HIV for a long time, and most of them have also been exposed to no therapy or to more toxic therapy than the used nowadays.

Likely the cause of the NI in HIV individuals is not monocausal and the accumulation of several facts occurred during the lifetime might be impacting in the neurocognitive function. However, the immune damage induced by HIV and its consequences seems to be relevant among these potential causes.

We also performed a geriatric assessment and we observed how comorbidity is associated with positive screening for NI. A trend was observed in those patients with medium–low comorbidity levels in the Charlson Comorbidity index, where higher scores were related to positive screening in the MOCA test. Among comorbidities, diabetes was found to be more represented in the positive NI screening group [19]. As previously reported, several studies revealed that sustained levels of hyperglycemia, poor glycemia controls and insulin resistance could influence the development of neurocognitive disorders [19, 32, 33].

Our study has some limitations, since this is a reduced sample size single center study. Moreover, the COVID-19 pandemic made us cancel the activity of the HIV-frailty clinic. However, this study has also some strengths, because all individuals included are well characterized with a multidisciplinary approach, where different specialists assessed all the included individuals.

## Conclusions

One third of individuals tested positive for the NI screening in our cohort. According to our results, those individuals with a nadir CD4+ T-cell count < 350 cells/mm^3^, lower education level and the presence of comorbidities such as diabetes mellitus had an increased risk of developing NI and should be prioritized in neurocognitive assessment among HIV older adults.

## Data Availability

Data of this study are not public, but are available under reasonable request to the corresponding author.
